# 12. Development of Provider-Specific Antibiotic Prescribing Feedback for Inpatient Antibiotic Stewardship Programs in Veterans Affairs (VA) Facilities (ASP)

**DOI:** 10.1093/ofid/ofab466.012

**Published:** 2021-12-04

**Authors:** Matthew B Goetz, Matthew B Goetz, Michelle Fang, Feliza Calub, Pamela Belperio, Vanessa W Stevens, Lauri Hicks, Arjun Srinivasan, Arjun Srinivasan, Barbara E Jones, Melinda M Neuhauser, Makoto M Jones

**Affiliations:** 1 VA Greater Los Angeles Healthcare System and David Geffen School of Medicine at UCLA, VA-CDC Practice-Based Research Network, Los Angeles, California; 2 VA San Diego Healthcare System, San Diego, CA; 3 VA Greater Los Angeles Healthcare System, Los Angeles, California; 4 VA Salt Lake City Health Care System, Salt Lake City, Utah; 5 Centers for Disease Control and Prevention, Atlanta, Georgia; 6 University of Utah Health, Salt Lake City, UT; 7 Salt Lake City VA/University of Utah, Salt Lake City, Utah

## Abstract

**Background:**

Provision of provider-specific outpatient antibiotic prescribing data has resulted in significant decreases in antibiotic use. We describe the development of reports of inpatient antibiotic prescribing by hospitalists attending on acute medical wards in VA medical facilities.

**Methods:**

We created algorithms for determining the attending physician responsible for patient days present (DP), by considering changes of service (e.g., prior to admission from the emergency department) and transfers between services or physicians. Each antibiotic dose was assigned to a single attending, ward location, and service according to denominator assignment. Antibiotic use was grouped into Centers for Disease Control and Prevention drug categories and expressed as antibiotic days of therapy (DOT) per 1000 DP. Data were obtained from the VA Corporate Data Warehouse. Algorithms were iteratively refined based on reviews of medical records from three VA medical centers and applied to acute care patients at a single site for 2018-2020.

**Results:**

In 2018-2020, 294 attendings oversaw acute inpatient care for >= 14 DP. 129 attendings with >= 300 DP oversaw 88.0% of all patient care and prescribed 87.6% of all antibiotics (480 DOT/1000 DP, IQR 375-559), 90.1% of broad-spectrum therapy for hospital-onset infections (55 DOT/1000 DP, IQR 31-72) and 88.3% of resistant Gram-positive therapy (70 DOT/1000 DP, IQR 39-89) in inpatient wards. The distribution of antibiotic use for acute care ward patients amongst these 129 staff is shown in the following figure.

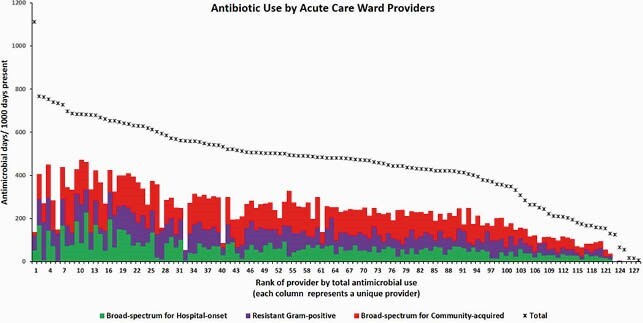

**Conclusion:**

We developed algorithms to attribute antibiotic therapy to inpatient attendings that can be broadly applied in facilities with electronic medical records. As with outpatient prescribing, we found large variation across inpatient attendings in overall antibiotic use and broad-spectrum antibiotic use. In future work, we will obtain provider feedback of report usability and interpretability and assess whether distribution of these reports allows antibiotic stewards to favorably influence provider prescribing practices.

**Disclosures:**

**Matthew B. Goetz, MD**, Nothing to disclose **Arjun Srinivasan, MD**, Nothing to disclose

